# A general nonaqueous sol-gel route to g-C_3_N_4_-coupling photocatalysts: the case of Z-scheme g-C_3_N_4_/TiO_2_ with enhanced photodegradation toward RhB under visible-light

**DOI:** 10.1038/srep39531

**Published:** 2016-12-22

**Authors:** Xu Liu, Nan Chen, Yuxiu Li, Dongyang Deng, Xinxin Xing, Yude Wang

**Affiliations:** 1 School of Materials Science and Engineering, Yunnan University, 650091 Kunming, People’s Republic of China; 2 Department of Physics, Yunnan University, 650091 Kunming, People’s Republic of China; 3 Yunnan Province Key Lab of Micro-Nano Materials and Technology, Yunnan University, 650091 Kunming, People’s Republic of China

## Abstract

The g-C_3_N_4_-coupling TiO_2_ photocatalysts with controllable particle size as well as the interface contact were prepared by a general nonaqueous sol-gel method. The structural and morphological features of g-C_3_N_4_/TiO_2_ were investigated through the X-ray diffraction, Fourier transformed infrared spectra, scanning electron microscopy and transmission electron microscopy, respectively. It is found the TiO_2_ nanoparticles with a size of 7.3 ± 1.6 nm are uniformly anchored on the surface of the g-C_3_N_4_ nanosheets in isolation. The photocatalytic properties of as-prepared g-C_3_N_4_/TiO_2_ were tested by degradation of Rhodamine B (RhB) under visible light, and an enhanced activity is observed. The mechanism of the enhanced activity was further investigated through N_2_ adsorption-desorption isotherms, UV-vis spectra, photoluminescence spectra, photoelectrochemical measurements, radical trapping experiments and X-ray photoelectron spectroscopy. Furthermore, the photocatalytic performances of obtained g-C_3_N_4_/TiO_2_ under sunlight were also evaluated in aspects of degradation efficiency and stability. The results indicate that the obtained g-C_3_N_4_/TiO_2_ is one promising photocatalyst for practical applications. The study of as-prepared g-C_3_N_4_/TiO_2_ also implies that the present method could be a general route of g-C_3_N_4_-coupling photocatalysts.

Facing energy depletion and environment pollution, the techniques utilizing inexhaustible sunlight to catalyze specific reactions, such as hydrogen production from water splitting^1^,^2^,^3^, CO_2_ reduction into hydrocarbon fuels^4^,^5^, as well as decomposition of environmental pollutant attract numerous attentions^6^,^7^,^8^,^9^,^10^. For these applications, the core is highly active photocatalysts which possess similar operation mechanisms including formation of photo-generated carries and corresponding redox reactions^11^. Due to the mechanisms, the photocatalysts suffer from some inherent weaknesses. Specifically, wide-band catalysts can only utilize ultraviolet which only takes lower than 6% of the sunlight; visible-light catalysts possesses low redox ability; both of them surfer from the recombination of the photogenerated carries. One of the effective solutions is semiconductor coupling which seems a general route to get over the inherent weaknesses for both wide-band and visible-light photocatalysts^12^. Through typical heterojunction charge transfer, the coupling of visible-light and wide-band photocatalysts endows wide-band catalyst better utilizations of visible light. On the other hand, through Z-scheme charge transfer, the coupling of visible-light photocatalysts can also realize stronger redox ability. No matter Z-scheme or typical heterojunction charge transfer, the possibility of carries recombination will be decreased. However, the formation of the semiconductor coupling faces two issues: choosing proper semiconductor components with suitable band structure, and projecting effective preparation methods to couple the components.

Graphitic carbon nitride (g-C_3_N_4_) is a polymeric semiconductor which consists of two earth-abundant elements (carbon and nitrogen), and easy to be prepared through thermal condensation of several low-cost nitrogen-rich precursors. It possesses a low band gap of 2.67 eV and relative more negative CB position of −1.1 eV^13^,^14^,^15^. Such the band structure makes g-C_3_N_4_ suitable to form semiconductor coupling with both wide-band and visible-light semiconductors. For wide-band semiconductor, the narrow band gap of g-C_3_N_4_ induces better absorption of visible light, and the relative negative CB position guarantees the typical heterojunction charge transfer under visible light. For visible-light semiconductor, the relative more negative CB position results in a high possibility of forming Z-scheme charge transfer, and g-C_3_N_4_ acts as reduction site. Moreover, the polymeric nature of g-C_3_N_4_ endows sufficient flexibility which leads that g-C_3_N_4_ can serve as an anchoring support for various inorganic nanoparticles. Owing to these extraordinary characters, g-C_3_N_4_-based photocatalyst has gained increasing investigation which naturally includes g-C_3_N_4_-coupling photocatalysts, and several g-C_3_N_4_-based semiconductor coupling systems with enhanced photocatalytic activity have been reported^16^,^17^,^18^. However, because the calcinations process is necessary in the preparation of g-C_3_N_4_, most of the reported literatures use two-step mechanical mixing or one-pot calcinations methods to prepare g-C_3_N_4_-coupling photocatalyst^19^,^20^,^21^,^22^. Obviously, the aforementioned methods are hard to achieve a well-dispersed structure on the surface of g-C_3_N_4_, controlled particle size as well as fine tuning of the interface contact, which greatly hinder the catalytic performance^23^. Considering the wide use of g-C_3_N_4_-coupling photocatalysts, it is meaningful to search a proper preparation method to obtain well-controlled morphology, especially a general approach through which a series of g-C_3_N_4_-coupling photocatalysts can be successfully prepared. To this task, one desire way is combining g-C_3_N_4_ with a well-investigated method which is a general route to nanostructure semiconductors.

When it comes to a general route to nanostructure semiconductors, the surfactant-free nonaqueous sol-gel methods that only involve organic solvents and metal organic precursors in the reaction system can not be ignored^24^. Among the organic solvents used in surfactant-free nonaqueous methods, benzyl alcohol and its derivatives, first reported by Niederberger *et al*., are the most brilliant^25^,^26^. Through the versatile solvents, numerous semiconductors including nitrides^27^,^28^, sulfides^29^, binary and ternary oxides^30^,^31^ have been effectively synthesized. It covers most of the semiconductors used in heterogeneous photocatalysis. More importantly, its products are easy to be controlled in nanoscale, even smaller than 10 nm, with few aggregations, which is what we want in preparation of g-C_3_N_4_-coupling photocatalysts. On the other hand, Pinna’s group found that graphene oxide is compatible with the “benzyl alcohol route”, and a series of graphene/metal oxide heterostructures like SnO_2_^32^, Fe_3_O_4_^33^, as well as TiO_2_^34^ based graphene heterostructures were obtained. For the obtained heterostructures, the oxide nanoparticles with ultrasmall size within 10 nm are uniformly anchored on the surface of reduction graphene oxide.

As we know, g-C_3_N_4_ possesses the similar layered structure with graphite. The layered g-C_3_N_4_ nanosheets could be also compatible with the “benzyl alcohol route”, which could be a general route to g-C_3_N_4_-coupling photocatalysts with controlled particle size as well as fine tuning of the interface contact. To our best knowledge, such the combination is still unreported. Meanwhile, TiO_2_ is the first reported oxide through the “benzyle alcohol route”, and its preparation mechanism and size tuning method have been investigated in detail^35^,^36^,^37^. Zhu *et al*. also reported that the TiO_2_ obtained through such the “benzyl alcohol route” shows good photodegradation performance toward phenol^38^. Choosing the mature components can greatly decrease the investigation difficulty in the compatibility.

Hence, this work uses g-C_3_N_4_/TiO_2_ as the case to verify whether g-C_3_N_4_ is compatible with the “benzyl alcohol route” to fabricating g-C_3_N_4_-coupling photocatalysts. The crystal structure and morphology features of obtained g-C_3_N_4_/TiO_2_ were fully characterized. The coupling sample shows double degradation efficiency of Rhodamine B under visible light than either g-C_3_N_4_ or TiO_2_. Its Z-scheme enhanced mechanism was investigated through radical scavenger tests, X-ray photoelectron spectroscopy (XPS) and UV-vis spectrum. Moreover, a good performance including degradation efficiency and photocatalytic stability of the photocatalyst under sunlight was also observed.

## Results and Discussion

### Structure and morphology of as-prepared g-C_3_N_4_/TiO_2_

**Figure 1:**
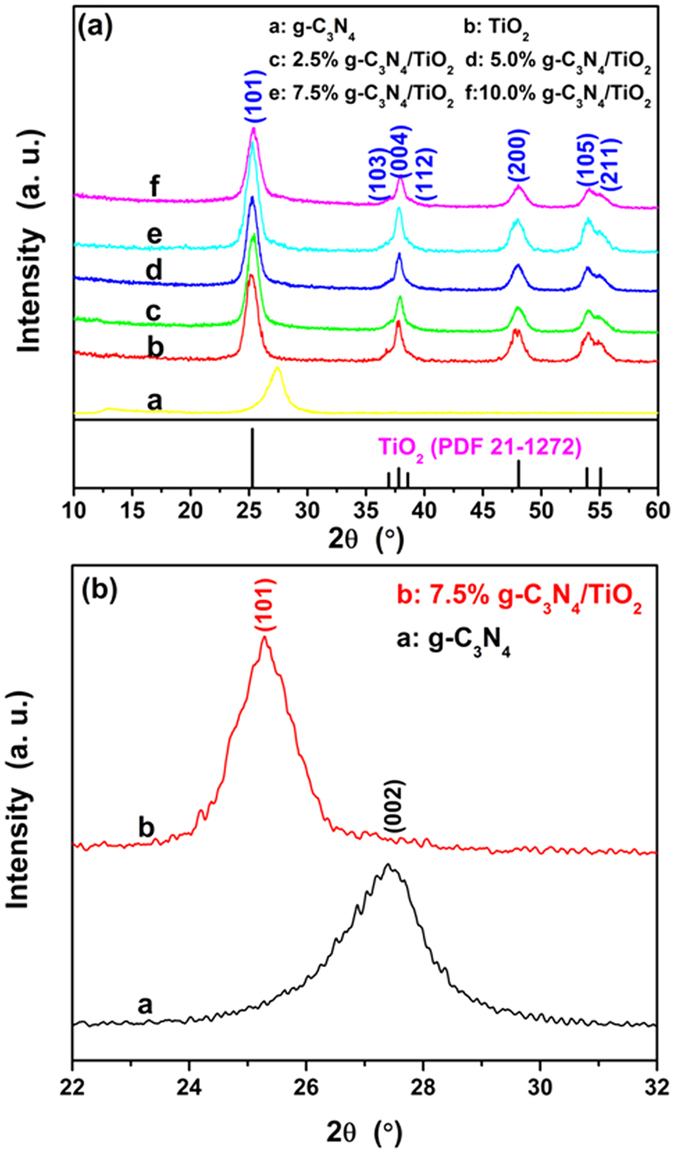
XRD patterns of (**a**) as-prepared samples and (**b**) enlarged view of the 22°–32° region.

The crystal structures of as-prepared samples were characterized by XRD, and the experimental data are shown in Fig. 1. For as-prepared pure g-C_3_N_4_, the curve exhibits a typical XRD pattern of g-C_3_N_4_, which consists of two characteristic peaks around 13.04° of (100) crystal plane, and 27.36° of (002) crystal plane^39^,^40^,^41^, respectively. All the peaks of pattern b match well with the (101), (103), (004), (112), (200), (105) and (211) reflections of anatase-type TiO_2_ (PDF No. 21-1272). The other patterns display the XRD curves of g-C_3_N_4_/TiO_2_. Comparing them with that of TiO_2_ and g-C_3_N_4_, one can find that the major peaks are identical to that of TiO_2_, including the shape and position. It seems the relative amount of g-C_3_N_4_ is very low so that only a small peak at 27.36°, the (002) reflection of g-C_3_N_4_, is observed, as shown in Fig. 1(b). The results indicate that the added g-C_3_N_4_ does not influence the formation of TiO_2_. On the other hand, it is observed that the peaks belonging to TiO_2_ possess an observed diffraction widening, so as to the overlapping of some adjacent peaks, such as the overlapping of (103), (004) and (112) peaks. These phenomena imply the TiO_2_ ought to have a small particles size which is an important parameter determining the photocatalytic activity. Rietveld refinement was used to have further revealing of the structural characters of TiO_2_. The refined pattern together with the experimental data, Bragg position as well as difference curve are shown in Fig. S1. One can find that the black calculated pattern matches well with the experimental data and the difference curve only shows fluctuations, indicating that the calculated value is convinced. Through the refinement, the grain size of the TiO_2_ is calculated to be 7.65 nm. Such the ultra-small particle size is identical to the reports of such the “benzyl alcohol route”. Apart from the grain size, the other structural parameters including the space group, cell parameters, atom coordinate as well as microstrain are listed in Table S1.

**Figure 2:**
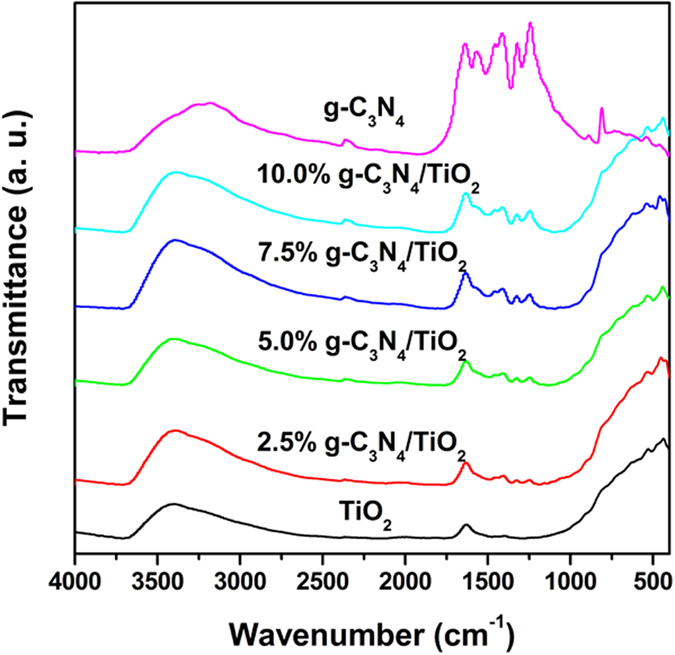
FTIR spectra of as-prepared samples.

Due to the overlapping and widening of the peaks, the XRD peaks of g-C_3_N_4_ are undistinguished in the pattern of as-prepared g-C_3_N_4_/TiO_2_. In this case, the FTIR was carried out to further confirm the structure of the g-C_3_N_4_/TiO_2_, and the results are shown in Fig. 2. Compared with that of TiO_2_, a series of peaks in the range of 1700–1200 cm^−1^ is easily observed in the pattern of g-C_3_N_4_, and belongs to the typical stretching modes of CN heterocycles. These peaks are also can be found in the curves of g-C_3_N_4_/TiO_2_ samples. Their intensity increases with the amount of g-C_3_N_4_. The results demonstrate that the structure of g-C_3_N_4_ is remained after the growth of TiO_2_.

**Figure 3:**
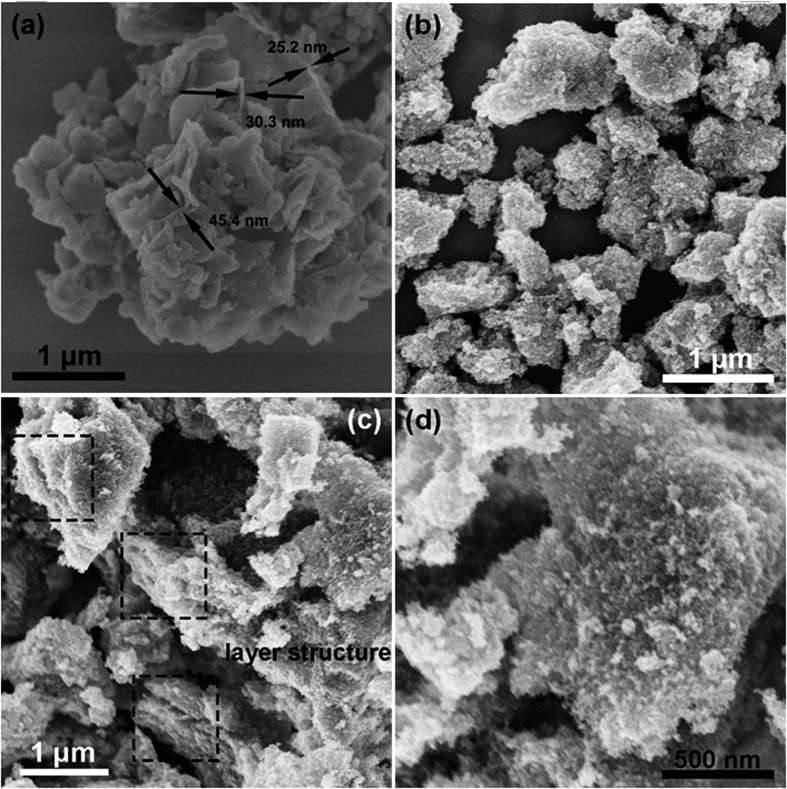
FESEM imagines of as-prepared samples. (**a**) g-C_3_N_4_, (**b**) TiO_2_, (**c**) and (**d**) 5% g-C_3_N_4_/TiO_2_.

To observe the micro-morphology of as-prepared samples, FESEM was carried out. Figure 3(a) displays the morphology of g-C_3_N_4_. It can be seen that the g-C_3_N_4_ is made up of stacking nanosheets whose thickness is easy to be measured and falls in tens nanometers. The surface morphology of TiO_2_ and 5% g-C_3_N_4_/TiO_2_ is shown in Fig. 3(b) and (c) which have the same magnification as Fig. 3(a). In Fig. 3(b), several submicron-size aggregations of TiO_2_ nanoparticles are observed. Under the same magnification, the morphology of g-C_3_N_4_/TiO_2_ is different from that of g-C_3_N_4_ and TiO_2_. The surface of the composite is rough as that of TiO_2_, but the size of the composite is much larger than that of TiO_2_ aggregations. For a better observation of the finer morphology, the surface of g-C_3_N_4_/TiO_2_ is magnified and shown in Fig. 3(d). The finer structure consists of ultra-small particles. Combined the experimental section as well as the XRD results, it is believed that the ultra-small nanoparticles might belong to TiO_2_ introduced by the benzyl alcohol reaction. Besides, the layered structure belonging to g-C_3_N_4_ is also can be seen in the region marked by square in Fig. 3(c). The results imply that the morphology may result from that the TiO_2_ nanoparticles are tightly anchored on the surface of g-C_3_N_4_. The SEM imagines of 2.5%, 7.5% and 10% g-C_3_N_4_/TiO_2_ are shown in Fig. S2. The similar morphology can be observed in all these samples.

**Figure 4:**
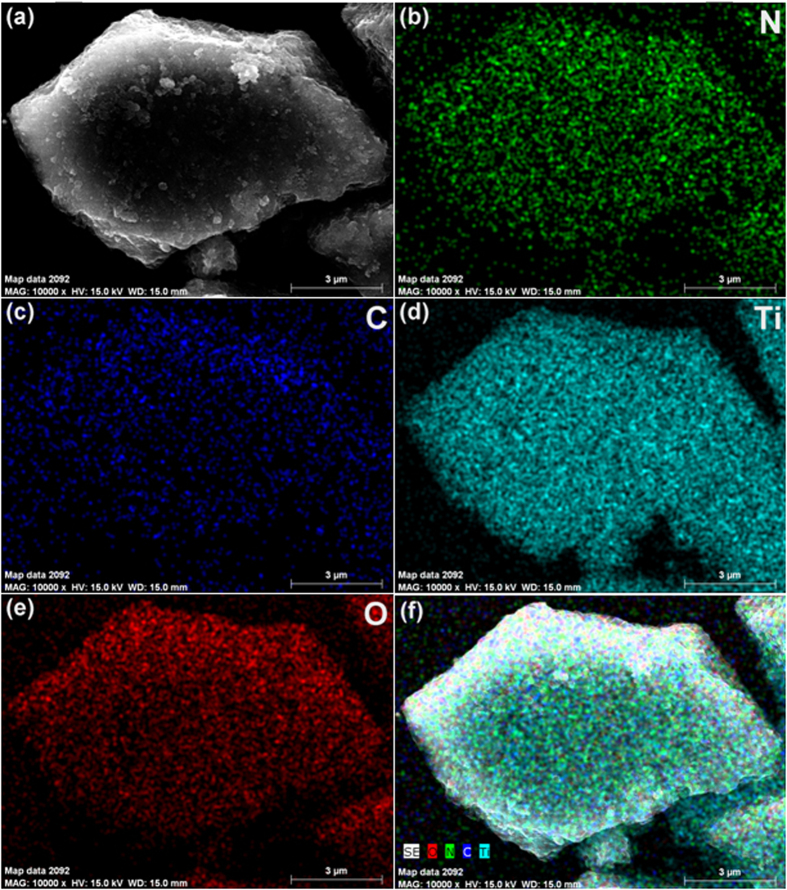
FESEM imagine of as-prepared g-C_3_N_4_/TiO_2_ and corresponding EDX mapping images of N, C, Ti and O.

To verify the point that the TiO_2_ is anchored on the surface of g-C_3_N_4_, element mapping of the g-C_3_N_4_/TiO_2_ is carried out and its results are shown in Fig. 4. Figure 4(a) displays the SEM image of the g-C_3_N_4_/TiO_2_ composite whose edge shows the layered structure. As shown in Fig. 4(b,c,d) and (e), N, C, Ti, O uniformly distribute on the region of the composite, even in the edge region, which fully proves the prediction about the distribution of g-C_3_N_4_ and TiO_2_ in the composite. In Fig. 4(f), it is also observed that the signal of Ti and O is much stronger than that of C and N. This phenomenon can be explained by the lower content of g-C_3_N_4_ in the composite.

**Figure 5:**
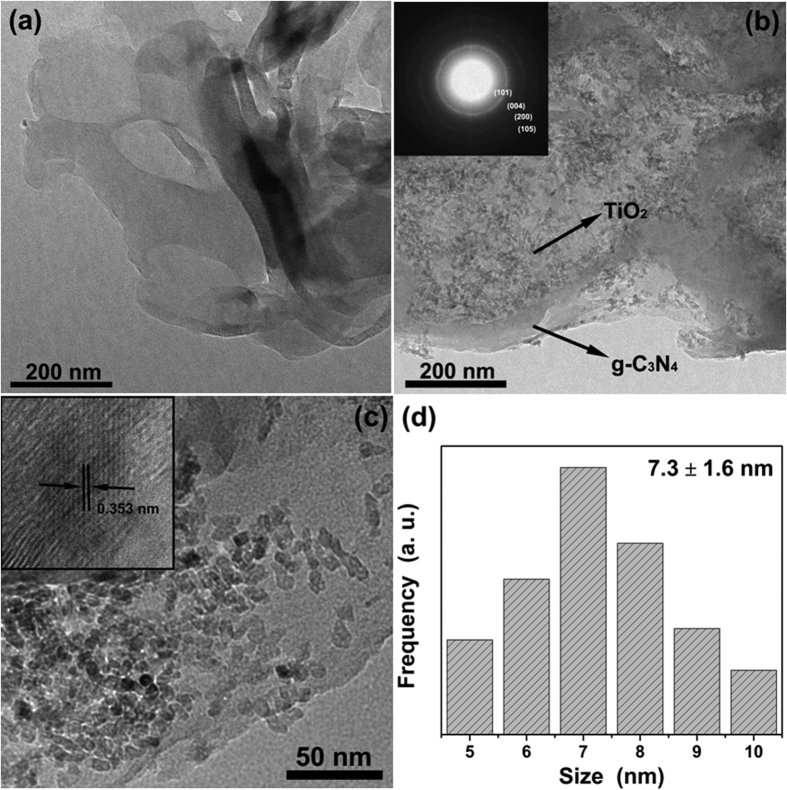
TEM imagines of as-prepared (**a**) g-C_3_N_4_ and (**b**) 7.5% g-C_3_N_4_/TiO_2_. (**c**) Magnified TEM imagine of as-prepared g-C_3_N_4_/TiO_2_. (**d**) Grain size distribution of TiO_2_ nanoparticles anchored on the surface of g-C_3_N_4_. The insets of (**b**) and (**c**) correspond to the SAED pattern of 7.5% g-C_3_N_4_/TiO_2_ and HRTEM of one isolated TiO_2_ nanoparticle anchored on the surface of g-C_3_N_4_, respectively.

Due to the limited magnification of SEM, the morphology of the anchored TiO_2_ particles is not clear. Hence, TEM were carried out to gain a better understanding of the morphological and structural features. Figure 5(a) and (b) display the TEM imagines of g-C_3_N_4_ and 7.5% g-C_3_N_4_/TiO_2_, respectively. Comparing these two figures, the similar results with that of FESEM can be observed. The g-C_3_N_4_ is made up of the stacking thin nanosheets whose surface is rather smooth. But it is clearly observed the surface of g-C_3_N_4_ is full of tens thousands of ultrasmall nanoparticles. With the selected-area electron-diffraction (SAED), a series of clear diffraction rings corresponding to the reflections (101), (004), (200) and (105) of anatase-type TiO_2_ is observed in the inset, which further reveals that the anchored nanoparticles are polycrystal anatase TiO_2_. Figure 5(c) exhibits the magnified TEM imagine of g-C_3_N_4_/TiO_2_. The well-define TiO_2_ nanoparticles are randomly anchored on the surface of g-C_3_N_4_, and no hard aggregation is observed between the nanoparticles. The tight combination between g-C_3_N_4_ and TiO_2_ is favorable to charge transfer between these two semiconductors which possess the proper band structure. While the uniform and isolated distribution of the TiO_2_ nanoparticles is beneficial for exposing more active site for the heterogeneous catalysis. The inset displays the HRTEM imagine of one isolated TiO_2_ nanoparticle. The clear fringes make it easy to estimate the interplanar spacing which is 0.353 nm and corresponding to the (101) plane of anatase. On the other hand, since the anchored nanoparticles show a well-defined morphology, the grain size of these particles are obtained by measuring the size of five hundreds particles, and the results are shown in Fig. 5(d). The result, 7.3 ± 1.6 nm, matches well with refinement of XRD. Both of them indicate that the TiO_2_ on the surface of g-C_3_N_4_/TiO_2_ prepared through the “benzyl alcohol route” possesses an ultrasmall particle size.

With the XRD, FTIR, FESEM, element mapping and TEM toward the as-prepared samples, it is sure that g-C_3_N_4_ is compatible with the “benzyl alcohol route” in the term of crystal structure and morphology. The addition of g-C_3_N_4_ does not disturb the chemical reactions and crystallization in the nonaqueous sol-gel route based on benzyl alcohol. Moreover, the formed TiO_2_ nanoparticles with ultrasmall grain size are uniformly anchored on the surface of g-C_3_N_4_ without hard aggregations in a wide range. Such the scenario is exactly what we want and seldom reported before. And such the good combination of TiO_2_ and g-C_3_N_4_ can be attributed three main reasons: (1) the “benzyl alcohol route” gives the TiO_2_ a very small particle size; (2) the electronegativity of the g-C_3_N_4_ nanosheets endows a strong electrostatic adsorption of Ti^4+^, which leads to a further tight combination^42^; (3) more importantly, g-C_3_N_4_ nanosheets possesses very good compatible with the “benzyl alcohol route” like the GO does.

### Photocatalytic properties of as-prepared g-C_3_N_4_/TiO_2_ under visible light

**Figure 6:**
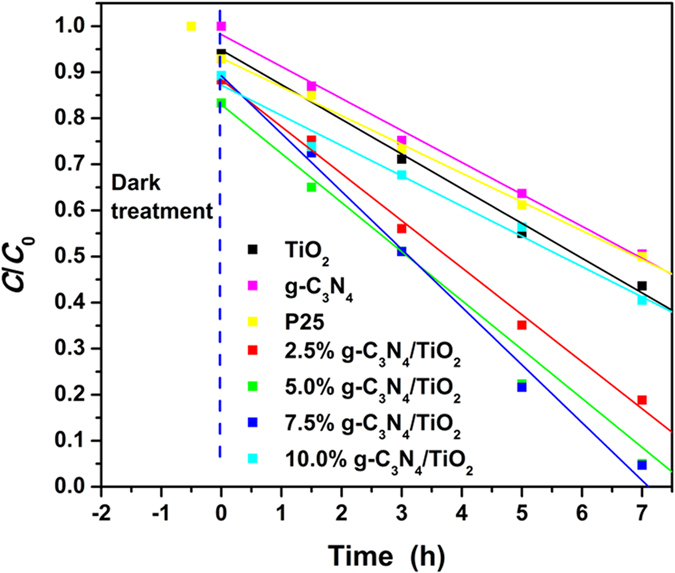
Photocatalytic activity plots of as-prepared samples for degradation of RhB under visible light.

It has been proved that g-C_3_N_4_ is compatible with the “benzyl alcohol route” to prepare g-C_3_N_4_/TiO_2_ nanocomposites from the aspects of structure and morphology. In this section, the photocatalytic properties of as-prepared g-C_3_N_4_/TiO_2_ under visible light are evaluated. And RhB, a typical organic dye which is a common water pollutant and could cause long-term environmental toxicity and short-term public health damage^43^,^44^,^45^, is chosen as the model pollutant. Prior the irradiation under the light source, half hour dark treatment was carried out to realize adsorption equilibrium and eliminate the influence of adsorption on degradation. Figure 6 shows the evolution of degradation rate along with the time. All the curves have a similar linear downtrend which seems to be zero-order kinetic process. Hence, the data points after the dark treatment were fitted according to the zero-order kinetic process:


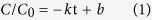


where *k* is the degradation rate constant, and *b* represents the residual composition at 0 h. The *k*, *b*, and *R*^2^ values of the fitted curves for all samples are listed in Table 1. Firstly, all the *R*^2^ values higher than 0.9842 indicating the fitted curves match well with the obtained data, and the degradation surely follows the zero-order kinetic process. Secondly, the as-prepared g-C_3_N_4_/TiO_2_ nanocomposites show higher degradation rate constant than both TiO_2_ and g-C_3_N_4_, and even the commercial P25 photocatalyst, which demonstrates a high photocatalytic activity of as-prepared g-C_3_N_4_/TiO_2_ nanocomposites. The compounding of g-C_3_N_4_ and TiO_2_ through combining g-C_3_N_4_ and “benzyl alcohol route” is meaningful. Thirdly, the composite containing 7.5% g-C_3_N_4_ displays the highest *k* value, which means 7.5% is the best ratio of g-C_3_N_4_ in the presented preparation method. On the other hand, the *b* in the fitted equation can reflect the adsorption ability of the photocatalyst, because it represents the residual composition at 0 h and after dark treatment. From the listed *b* values in Table 1, the as-prepared g-C_3_N_4_/TiO_2_ nanocomposites have the lower value than as-prepared g-C_3_N_4_ and TiO_2_. Such the stronger adsorption ability can be understood from the FESEM and TEM imagines. The morphology that the ultrasmall TiO_2_ nanoparticles uniformly anchored on the surface of g-C_3_N_4_ prevents the aggregations of TiO_2_ and increase the surface roughness of g-C_3_N_4_. But for the g-C_3_N_4_/TiO_2_ with different content of g-C_3_N_4_, the values of *b* are random which indicates that the enhanced activity is not only resulted from the better adsorption. For example, the sample with 7.5% g-C_3_N_4_ possesses the best degradation efficiency, but its *b* value is the highest among the other samples of g-C_3_N_4_/TiO_2_. Hence, it is believed that there are some other reasons leading to the enhanced activity apart from the better adsorption. In the introduction, it is predicted that the coupling of g-C_3_N_4_ and wide-band semiconductor could result in a better utilization of visible light, and separation as well as transfer of photogenerated carries. All of them could enhance the visible-light driven photocatalysis activity.

### Mechanisms of the enhanced photocatalytic activities of g-C_3_N_4_/TiO_2_

In this section, the mechanisms of the enhanced photocatalytic activities of g-C_3_N_4_/TiO_2_ were studied from the adsorption, utilization of visible light, and charge transfer, respectively.

**Figure 7:**
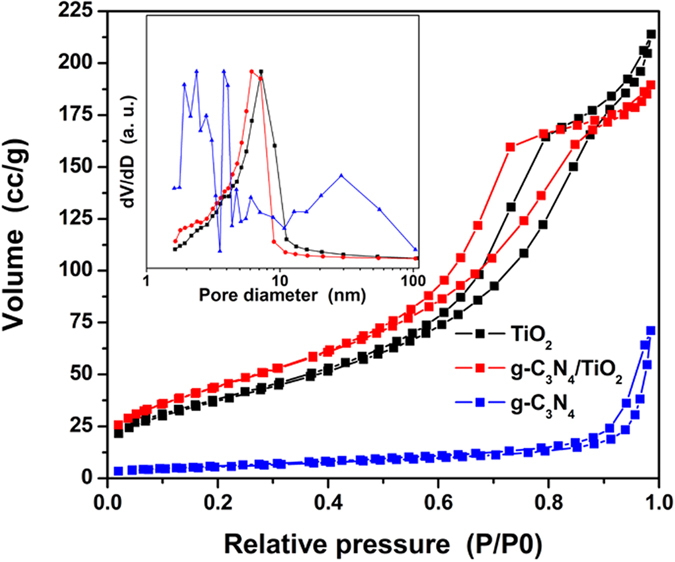
N_2_ adsorption-desorption isotherms of as-prepared TiO_2_, g-C_3_N_4_ as well as g-C_3_N_4_/TiO_2_, and the corresponding normalized pore-size distribution (inset).

In usual, a better adsorption is linked to a higher specific surface area^46^, so the N_2_ adsorption-desorption experiments toward the as-prepared TiO_2_, g-C_3_N_4_ and g-C_3_N_4_/TiO_2_ were carried out. Since the content of g-C_3_N_4_ is relative low, and there are four samples with different content, the 10% g-C_3_N_4_/TiO_2_ with highest amount of g-C_3_N_4_ was chosen as the example to make the results clearer and easier to be analyzed. The isotherms and corresponding pore size distribution curves are shown in Fig. 7. In the similar isotherms of TiO_2_ and g-C_3_N_4_/TiO_2_, the evident hysteresis loops from 0.5 to 0.9 can be observed, indicating the presence of mesopores within the materials. This can be further verified from the pore size distribution curves. The TiO_2_ and g-C_3_N_4_/TiO_2_ show the pores centered at 7.211 and 6.15 nm, respectively. According to the shape of the hysteresis loops and the TEM results, the observed mesoporous is resulted from the accumulation of TiO_2_ nanoparticles. The isotherm of g-C_3_N_4_ also shows non-closed and porous character as shown in the Fig. 7 and its inset. But its specific surface area is much lower than that of TiO_2_ and g-C_3_N_4_/TiO_2_. To be more specific, the value from BET measurement of TiO_2_, g-C_3_N_4_/TiO_2_ and g-C_3_N_4_ are 141, 166 and 21 m^2^ g^−1^, respectively. These results well prove the prediction made from the *b* values listed in Table 1.

**Figure 8:**
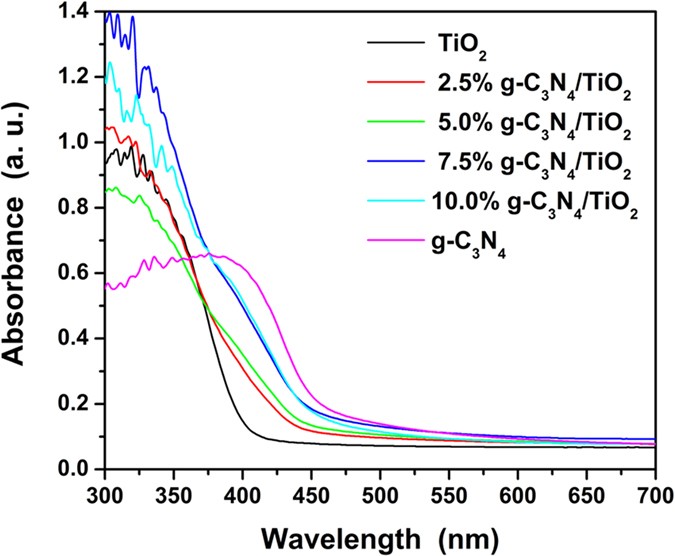
UV-vis spectra of as-prepared samples.

Figure 8 displays the UV-vis spectra of as-prepared samples. The curve of TiO_2_ displays an absorption edge in ultraviolet region, which corresponds to the band gap of TiO_2_. But the as-prepared TiO_2_ still shows some absorption in visible light. Such the absorption backs the visible-light driven photocatalytic activity of TiO_2_. Due to the narrower band gap of g-C_3_N_4_ (2.67 eV), the edge absorption of g-C_3_N_4_ arise at the region with longer wavelength than TiO_2_. Comparing the curves of g-C_3_N_4_/TiO_2_ with that of TiO_2_, one can find a better absorption of visible light from 400 to 550 nm which matches well with the spectra of g-C_3_N_4_, and the absorption is increased with the content of g-C_3_N_4_. Hence, it is clear that g-C_3_N_4_ enhances the visible-light utilization of TiO_2_, owing to the narrower band gap of 2.67 eV.

**Figure 9:**
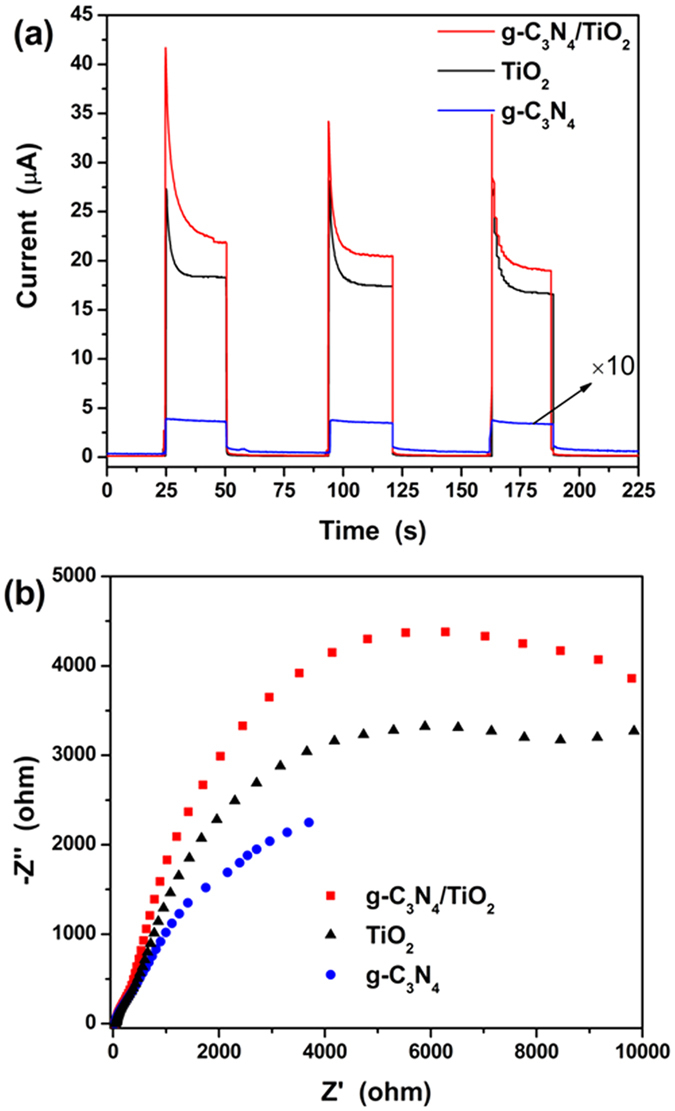
Photoelectrochmical measurement of TiO_2_, g-C_3_N_4_, and g-C_3_N_4_/TiO_2_ composite. (**a**) transient photocurrent responses and (**b**) EIS Nyquist plots.

It is generally accepted that the photoluminescence (PL) spectra can be used to check the separation and transfer of photogenerated carries^47^. Due to the separation and transfer, the recombination of the photogenerated carries are decreased, which further leads to reduced PL of the coupling. However, for the coupling of g-C_3_N_4_ and TiO_2_, such the method loses its function. Because the g-C_3_N_4_ shows very strong PL which even can be observed by naked eyes, but PL intensity of TiO_2_ is much lower. Hence, the composite shows stronger PL than TiO_2_, but lower PL than g-C_3_N_4_, as shown in Fig. S3. Such the result matches with other reported literatures^23^,^48^,^49^. In this situation, the photoelectrochemical measurement was carried out to verify the separation of the photogenerated carries. Figure 9(a) displays the transient photocurrent responses of TiO_2_, g-C_3_N_4_ and their composite through typical switch on-off cycles^50^,^51^,^52^. It is observed all the materials show fast response and recovery. And the composite shows the highest current, followed by TiO_2_. The lowest current of g-C_3_N_4_ may be attributed to the high recombination of the photogenerated carries which can be understood from Fig. S3. The highest photocurrent directly proves the separation of photogenerated carries in g-C_3_N_4_/TiO_2_. By the way, the electrochemical impedance spectroscopy (EIS) was carried out and its results are shown in Fig. 9(b). From the figure, the arc character of all samples can be observed. It is reported that the charge lifetime is related to the recombination resistance, and large semicircle radius of EIS curves implies large recombination resistance^53^,^54^,^55^. From the obtained EIS curves, one can find that the g-C_3_N_4_/TiO_2_ has the largest radius, while the g-C_3_N_4_ possesses the smallest. The results match well with that of photocurrents. From the photoelectrochmical measurement, it is known that the combination of g-C_3_N_4_ and TiO_2_ increase the recombination resistance and lifetime of photogenerated carries, which is resulted from the charge separation.

On the other hand, it is known to us that the reactive species trapping experiment is effective in judgment of active species. Further step, the mode of carries transfer as well as separation could be indirectly studied. Hence, it was carried out to study the possible separation and transfer of photogenerated carries. In this work, ethylenediamine teracetic acid disodium salt (EDTA-2Na, 0.01 M), *p*-benzoquinone (BZQ, 0.001 M) and isopropanol (IPA, 0.02 M) were used as scavengers for photogenerated holes, superoxide anion radicals and hydroxyl radicals, respectively^56^,^57^. The removal rates of the dye at 7 h with different catalysts were used for comparison. Because the behaviors of the composite highly depend on its components, and the function of the conduction band and valence band directly influence the judgment of the charge transfer model, the behavior of two components, TiO_2_ and g-C_3_N_4_, are firstly studied.

**Figure 10:**
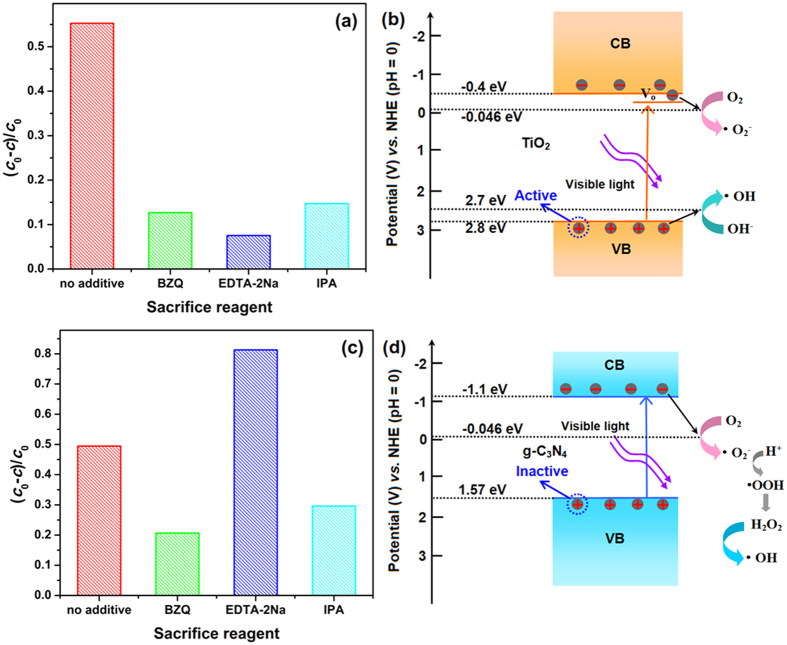
Reactive species trapping experiments of as-prepared (**a**) TiO_2_ and (**c**) g-C_3_N_4_. The degradation mechanisms of as-prepared (**b**) TiO_2_ and (**d**) g-C_3_N_4_. The pillars in (**a**) and (**c**) show the removal rate of the dye at 7 h.

Figure 10(a) displays the results of TiO_2_. Without any trapping reagent, the as-prepared TiO_2_ shows the ability of breaking down the RhB dye under visible light. With addition of the sacrifice reagents, the removal rates of the dye decrease, which means photogenerated holes, superoxide anion radicals and hydroxyl radicals are produced and active during the degradation process. Especially the active photogenerated holes exclude the dye-sensitization mechanism of the degradation, because the hole is not involved in the dye-sensitization process^58^. However, it is generally accepted that the band gap of anatase-type TiO_2_ is 3.2 eV which is too wide to produce photogenerated carries and consequent corresponding redox reactions. But the result of the trapping reagents indirectly proves the existence of aforementioned process. For these, we attribute these to the possibly existing oxygen vacancies in the TiO_2_ nanoparticles. Nakamura *et al*. created oxygen vacancies in TiO_2_ by plasma treatment to increase its photocatalytic activities under visible light, and brought out that the oxygen vacancies facilitate visible light absorption by generating discrete state below the conduction band of TiO_2_^59^. Ihara *et al*. also reported that oxygen vacancies can be easily created in the grain boundaries^60^. In this work, the TiO_2_ nanoparticles anchored on the surface of g-C_3_N_4_ have a very small grain size, and they are easy to form varies defects on their surface, naturally include the oxygen vacancy. It is believed that the oxygen-related defects including the oxygen vacancy can be indirectly observed in the O1s XPS spectra in the form of absorbed oxygen ion. To verify the existence of the absorbed oxygen ion, XPS toward TiO_2_ and 7.5% g-C_3_N_4_/TiO_2_ were carried out. The magnified O1s XPS spectra are shown in Fig. S4(a) and (b), respectively. The peak with lower binding energy around 530 eV is associated to the oxygen atom in the crystal lattice, while the peak with higher binding energy is assigned to the absorbed oxygen ion^61^. It proves that there surely exist the oxygen related defects including the oxygen vacancy. And it also forebodes that the TiO_2_ in the g-C_3_N_4_/TiO_2_ could also show the similar photocatalytic behaviors to the pure TiO_2_. Based on the oxygen vacancy introducing defect level and the results of sacrifice reagent, the degradation mechanism of as-prepared TiO_2_ under visible light is obtained as shown in Fig. 10(b).

Unlike wide-band TiO_2_, g-C_3_N_4_, a visible-light semiconductor with a narrow band gap of 2.67 eV, is easy to be excited by visible light. Its result of trapping experiments is shown in Fig. 10(c). Two unusual phenomena are observed, including that the addition of hole trapping reagent (EDTA-2Na) intensively increases the removal rate, and the hydroxyl radical arises and show its function in the degradation process. The active species in the degradation process can be predicted through comparing the top of valence band (TVB) potential and bottom of conduction band (BCB) potential with the standard redox potential (SRP) of corresponding reactions. Since the *E*_BCB_ and *E*_TVB_ of g-C_3_N_4_ have been widely studied, −1.10 eV and 1.57 eV are adopted to be the *E*_BCB_ and *E*_TVB_, respectively^16^. Also, it is known to us that the SRP of O_2_/·O_2_^−^ and OH^−^/·OH are −0.046 and +2.7 V *vs*. NHE, respectively^12^. Due to the *E*_BCB_ of g-C_3_N_4_ is more negative than the SRP of O_2_/·O_2_^−^, the photogenerated electrons can react with O_2_ to form ·O_2_^−^. But the *E*_TVB_ of g-C_3_N_4_ is lower than the SRP of OH^−^/·OH, so ·OH can not be formed through the reaction of photogenerated holes and OH^−^. Although the photogenerated holes can not produce ·OH, it is believed the generated holes still can oxidize the pollutant. So, it is generally accepted that the effective species during the photocatalysis reaction of g-C_3_N_4_ are holes, and ·O_2_^− ^62,63. According to the result shown in Fig. 10(c), the prediction can not explain the behavior of the g-C_3_N_4_ for the degradation of RhB. Therefore, the effect of the conduction band and valence band of g-C_3_N_4_ need further analysis. As we know, the potential of TVB and BCB decides the redox ability of the photogenerated carries. More negative BCB means stronger reduction photogenerated electrons, and more positive TVB means stronger oxidation photogenerated holes. The g-C_3_N_4_ possesses very negative BCB, but its TVB is weak. Hence, the oxidation ability of the photogenerated hole is weak which makes the hole show few effects on the RhB molecules just like the hole can not react with OH^−^ to form ·OH. Moreover, the excess photogenerated holes would recombine with the photogenerated electrons which can form the effective ·O_2_^−^ species. Especially, the g-C_3_N_4_ always shows a strong blue photoluminescence, indicating a strong recombination of the photogenerated carries. With the addition of EDTA-2Na, the trapping reagent of photogenerated holes, the inactive holes are consumed, and the reduced amount of generated holes decreases the recombination possibility of photogenerated carries. That is to say, more photogenerated electrons are kept on the conduction band, so the removal rate is increased. On the other hand, due to the strong reduction of the photogenerated electrons, the formed ·O_2_^−^ also possesses very strong reduction which could leads to the further reactions and formation of ·OH radicals through an intermediate of ·OOH^64^,^65^. That is why the addition of IPA, ·OH trapping reagent, causes the lower removal rate. Hence, the degradation mechanism of g-C_3_N_4_ toward RhB solution is summarized in Fig. 10(d). From the trapping experiments toward TiO_2_ and g-C_3_N_4_, it is found that the most difference between them during the degradation process under visible light in the aspect of effective species is that the photogenerated holes in the valence band of TiO_2_ is active, but inactive for g-C_3_N_4_. Such the conclusion is different from the common reported literatures in which the difference is thought to be whether there are OH^−^ species according the compassion of calculated potent ion of BCB and TVB with the SRP of corresponding reactions^21^,^49^,^63^. At the same time, the conclusion is very important to the analysis of charge transfer model and mechanism of enhanced photocatalytic activity of g-C_3_N_4_/TiO_2_ nanocomposite. Hence, the analysis of trapping experiments also should base on the experiments of its components rather than simple theoretical comparisons.

**Figure 11:**
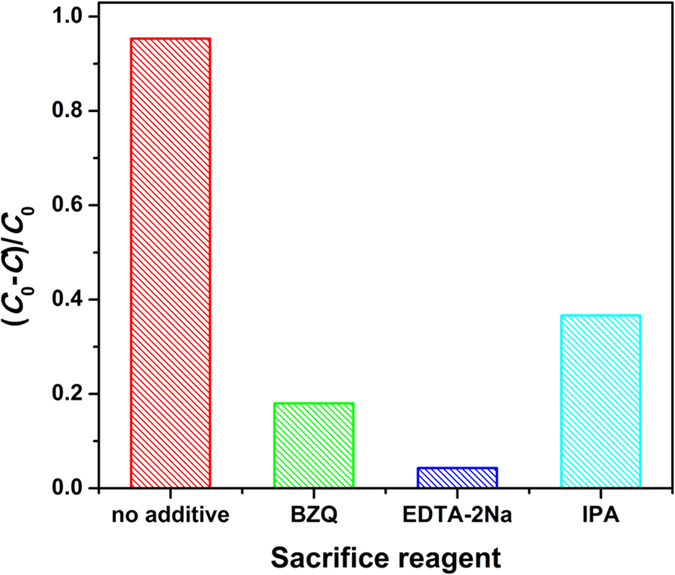
Reactive species trapping experiments of as-prepared 7.5% g-C_3_N_4_/TiO_2_. The pillars show the removal rate of the dye at 7 h.

The active specie trapping experiments toward 7.5% g-C_3_N_4_/TiO_2_ are carried out, and the results are shown in Fig. 11. General speaking, all three tested species including photogenerated hole, superoxide anion radical and hydroxyl radical are active during the photocatalysis process. Comparing with Fig. 10(a) and (c), one can find that the active species of the composite are different from that of g-C_3_N_4_, but similar to that of TiO_2_. Further step, the removal rate are normalized according to the removal rate with no additive. For pure TiO_2_, the normalized removal rate with BZQ, EDTA-2Na and IPA are 22.91%, 13.60%, and 26.59%, respectively. For 7.5% g-C_3_N_4_/TiO_2_, the normalized removal rate with BZQ, EDTA-2Na and IPA are 18.90%, 4.48%, and 38.45%, respectively. It is clear that the experimental data between TiO_2_ and 7.5% g-C_3_N_4_/TiO_2_ are distinguished, and it also could not be the simple superposition of g-C_3_N_4_ and TiO_2_. Especially for the removal rate with EDTA-2Na, the photogenerated hole is effective for TiO_2_, and inactive for g-C_3_N_4_. While for their composite it shows stronger effect than that for TiO_2_. Hence, the degradation model of g-C_3_N_4_/TiO_2_ is different from their components, which is resulted from the separation and transfer of the photogenerated carries.

**Figure 12:**
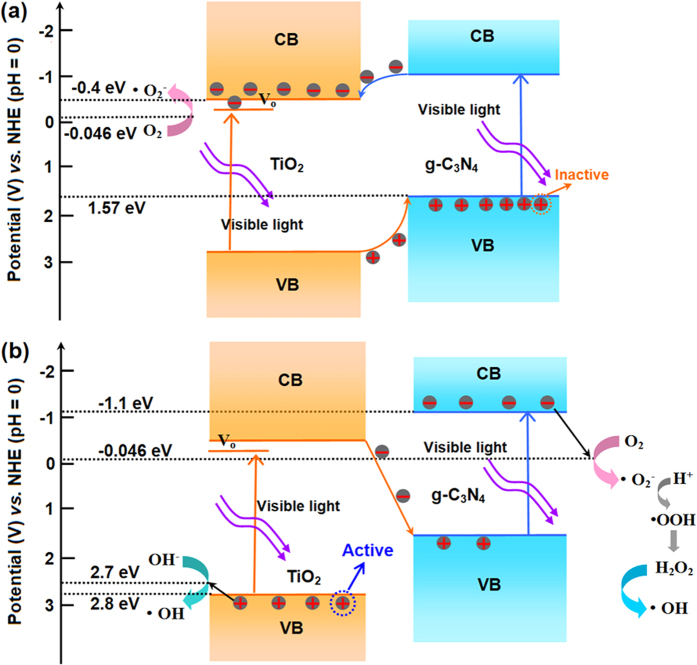
The degradation mechanisms of as-prepared g-C_3_N_4_/TiO_2_ in the form of (**a**) typical heterojunction and (**b**) Z-scheme.

On the other hands, according to the degradation mechanism of TiO_2_ (Fig. 10(b)) and g-C_3_N_4_ (Fig. 10(d)), the two possible separation and transfer model of carries for g-C_3_N_4_/TiO_2_ nanocomposite are shown in Fig. 12(a) and (b), which correspond to typical heterojunction and Z-scheme charge transfer models, respectively. Comparing these two models, one can find that the active species in the Z-scheme model are photogenerated hole, superoxide anion radical and hydroxyl radical, but the active species of heterojunction-type model only involves superoxide anion radical. For the heterojunction-type model, although it is not sure whether the superoxide anion radicals generated in the conduction band of TiO_2_ can produce hydroxyl radical, it is certain that the holes formed in the valence band of g-C_3_N_4_ is inactive. Comparing this certain difference between the two models with the experimental data, it can be concluded that the separation and transfer model of as-prepared g-C_3_N_4_/TiO_2_ is Z-scheme. Through the Z-scheme, the different behaviors of g-C_3_N_4_/TiO_2_ and TiO_2_ can be explained. The superoxide anion radical is generated on the conduction band of g-C_3_N_4_ whose *E*_BCB_ is more negative than that of TiO_2_. With stronger reduction, the effect of superoxide anion radical is enhanced compared with TiO_2_. Although the valence band is the place forming the active holes for both TiO_2_ and g-C_3_N_4_/TiO_2_, there is a lower possibility of carries recombination of g-C_3_N_4_/TiO_2_ than TiO_2_ due to the Z-scheme charge transfer in g-C_3_N_4_/TiO_2_. For hydroxyl radical, it is formed through the photogenerated holes on the valence band of TiO_2_. Since the photogenerated holes directly consumed by the dye molecules in g-C_3_N_4_/TiO_2_, the residual holes for generation of hydroxyl radical are decreased. Hence, the effect of hydroxyl radical is decreased for g-C_3_N_4_/TiO_2._ Through the trapping experiments, it proves the Z-scheme charge transfer between g-C_3_N_4_ and TiO_2_ in g-C_3_N_4_/TiO_2_. And the enhanced activity with the Z-scheme charge transfer can be attributed to the consequent stronger reduction ability of the material, and effective charge separation.

Hence, the enhanced photocatalytic activity of g-C_3_N_4_/TiO_2_ under visible light is resulted from the increased adsorption, better utilization of visible light, Z-scheme charge transfer as well as consequent stronger reduction ability toward the dye molecules.

### Photocatalytic performances of as-prepared g-C_3_N_4_/TiO_2_ under sunlight

In order to test the potential of g-C_3_N_4_/TiO_2_ in practical application, the photocatalytic performances of as-prepared g-C_3_N_4_/TiO_2_ nanocomposites under sunlight are tested. Because the 7.5% is the optimal content of g-C_3_N_4_, 7.5% g-C_3_N_4_/TiO_2_ was chosen as the object.

**Figure 13:**
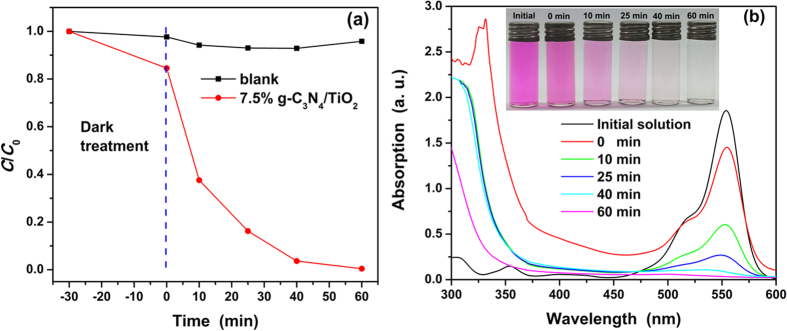
(**a**) Photocatalytic activity plots of as-prepared 7.5% g-C_3_N_4_/TiO_2_ for degradation of RhB under sunlight. (**b**) UV-vis spectra of the centrifuged solutions at different degradation time. Inset is the photo the centrifuged solutions.

Figure 13 displays the evolution of degradation rate along with the time. It is observed that the data points in the blank group fluctuates around 1, indicating that the degradation caused by the sunlight can be neglected during the test. With the addition of 7.5% g-C_3_N_4_/TiO_2_, the concentration of the RhB is decreased rapidly. It only takes 40 min to remove 96.37% RhB. 99.5% RhB was broken down at 1 h. Such the removal efficiency is about ten times faster than that under visible light. On the other hand, de-ethylation of RhB is always observed in its photodegradation, and the de-ethylation process could lead to the shift of absorption peak from 550 to 500 nm, and consequent decrease of absorbance at its initial position of absorption peak^66^. But the de-ethylation only takes a small part of the whole of degradation process, and double time is usually needed. In this term, the UV-vis spectra of the centrifuged solutions were tested and shown in Fig. 13(b). It is observed that the strong absorption peak has no shift during the degradation reaction. That is to say the rapid degradation of RhB pollutant is not resulted from the de-ethylation. Hence, the as-prepared 7.5% g-C_3_N_4_/TiO_2_ certainly possesses quite high photocatalytic activity. And the inset shows the photo of the centrifuged solutions. The color of the solution fades along with the time, and turns to be colorless and transparent solution. While the higher efficiency can be attributed to the different light resource: the first, the sunlight is much stronger than the 24 W lamp used as the visible-light resource; there are some ultraviolet in the sunlight, which is beneficial for exciting more carries for TiO_2_ component.

**Figure 14:**
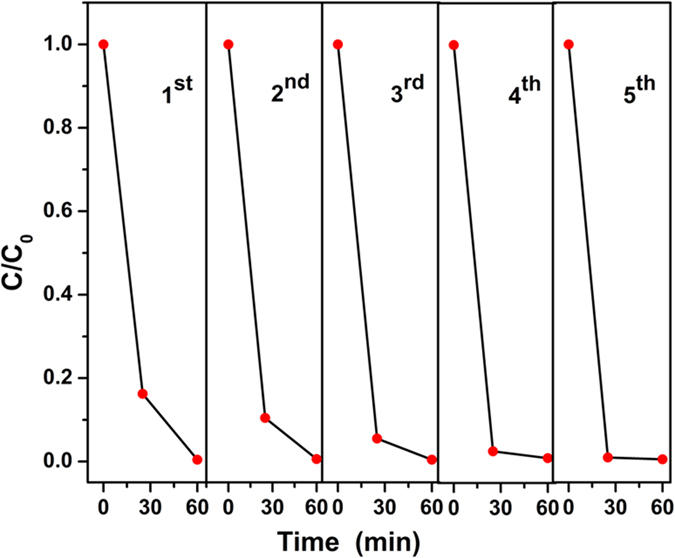
Repeated photocatalytic experiments of 7.5% g-C_3_N_4_/TiO_2_ for five times under irradiation of sunlight.

As we know, the photocatalytic stability of the catalysts is one of the most important parameter evaluating the possibility of practical application. Hence the degradation evolution in five cycles of degradation tests under sunlight was recorded as shown in Fig. 14. Unexpectedly, the photocatalyst not only keep a good stability, but also has a better efficiency with the increase of the cycle time. The degradation rate reaches higher than 99% for all the cycles at 60 min. At 25 min, the degradation rate reaches 83.77%, 89.55%, 94.48%, 97.40% and 99.02% for first, second, third, fourth and fifth cycle, respectively. Because the property is always associated with the microstructure of the catalysts, the TEM toward the catalyst after the 5 cycles was carried out, and shown in Fig. S5. In the Fig. S5, the composite of the sheet-like g-C_3_N_4_ and ultrasmall TiO_2_ nanoparticles are still observed. But the amount of the TiO_2_ nanoparticles loaded on the surface of g-C_3_N_4_ is much smaller than the primary sample shown in Fig. 5(b). The degradation efficiency is increased with the decreased amount of TiO_2_ loaded on the surface of g-C_3_N_4_ according to the results of TEM and stability tests. And Fig. 6 and Table 1 have proved that 7.5% is the best ration amount 0, 2.5%, 5.0%, 7.5% and 10.0%. Hence, the contradiction arises. Such the unusual phenomenon might be explained through the presented experiment details. In the preparation experiment, TiO_2_ is the matrix, and its amount is constant. The g-C_3_N_4_ is used as enhancer, and its amount is calculated according to the mass ratio of g-C_3_N_4_/TiO_2_. Such the design of the experiment ignores that although the g-C_3_N_4_ is compatible with benzyl alcohol, its dispersed amount is limited. The amount of g-C_3_N_4_ added in the preparation of 7.5% g-C_3_N_4_/TiO_2_ could be the maximum. With further addition of g-C_3_N_4_, it stacks with each other, and the exposed surface is limited for TiO_2_ to attach. Hence, 7.5% is the best in the projecting experiments. But the optimal ratio should be much higher than 7.5% so that the catalyst’s efficiency increases along with smaller loaded TiO_2_ nanoparticles. From this point of view, it suggests that the g-C_3_N_4_ should be used as the matrix or the baseline to calculate the amount of the other components. And the limited dispersing amount of g-C_3_N_4_ can be improved through decrease the thick of the g-C_3_N_4_ nanosheets, which also could make the “benzeyl alcohol route” more economic and the products more active. Anyhow, it has proven g-C_3_N_4_ is compatibility with the benzyl-alcohol-based nonaqueous sol-gel route, and the combination could be a general route to g-C_3_N_4_-coupling photocatalysts with controlled morphology and enhanced photocatalytic activity.

## Conclusions

Through the combination of g-C_3_N_4_ and classical nonaqueous sol-gel route based on benzyl alcohol, g-C_3_N_4_/TiO_2_ coupling photocatalysts were obtained. Owing to the contribution of benzyl-alcohol-based nonaqueous sol-gel route, the TiO_2_ on the surface of g-C_3_N_4_ shows ultrasmall grain size. Due to the excellent compatibility of g-C_3_N_4_ and the benzyl alcohol route, the TiO_2_ nanoparticles are uniformly distributed on the surface of g-C_3_N_4_. For the present experimental parameters, the optimal mass ratio of g-C_3_N_4_/TiO_2_ is 7.5%. And the enhanced photocatalytic activity under visible light is attributed to the Z-scheme heterojunction, and consequent better absorption of visible light, enhanced reduction ability as well as decreased recombination. The as-prepared g-C_3_N_4_/TiO_2_ also has high efficiency and good cycle performance under sunlight, indicating its practical application. Through the detailed study of as-prepared g-C_3_N_4_/TiO_2_ and considering that “benzyl alcohol route” is one general route to numerous size-controlled photocatalysts, it is believed that such the nonaqueous sol-gel route could be a general method to g-C_3_N_4_-coupling photocatalysts with desired morphology and contact interface.

## Methods

### Materials and synthesis

All the chemical reagents used in the experiments were obtained from commercial sources as guaranteed-grade reagents and used without further purification.

Well-defined g-C_3_N_4_ nanostructure was synthesized through thermal condensation of cyanuric acid-melamine complex^67^. Briefly, 6.45 g grinded cyanuric acid was added to 100 mL ethanol with stirring. 6.3 g melamine was then grinded and added to the above suspension. The mixture was continuing stirred for 8 h, and then dried at 60 °C. The obtained white powder was subsequently calcined at 550 °C under N_2_ atmosphere for 4 h with a heating rate of 2.3 °C min^−1^ to gain the final g-C_3_N_4_ powder.

The g-C_3_N_4_/TiO_2_ nanocomposites were *in-situ* prepared on the pre-synthesized nanostructured g-C_3_N_4_ through the “benzyl alcohol route”. Firstly, 10 mL xylol was poured into a closed conical flask, and then 0.69 mL TiCl_4_ was dissolved with magnetic stirring. Five minutes later, 66 mL benzyl alcohol was gently poured into the system. After stirring for another five minutes, the calculated amount of pre-synthesized g-C_3_N_4_ was further grinded and added to the titanium precursor solution. The amount of g-C_3_N_4_ was calculated according to mass ratios of g-C_3_N_4_/TiO_2_ = 0%, 2.5%, 5.0%, 7.5% as well as 10%, respectively. The corresponding samples were named as TiO_2_ and x% g-C_3_N_4_/TiO_2_ (x = 2.5, 5.0, 7.5, 10.0), respectively. To gain a better dispersing of g-C_3_N_4_ and promote the adsorption of Ti^4+^ on the g-C_3_N_4_ surface with electronegativity, the mixtures were treated with ultrasound for 2 h. Later, with further stirring for 30 s, 70 mL of the homogeneous precursors were transferred into the Teflon-lined stainless steel autoclaves with a capacity of 83 mL and reacted at 180 °C for only 4 h, respectively. The autoclaves were cooled down to room temperature, and the resulting precipitates were washed with ethanol for four times and dried at 60 °C overnight.

### Characterization of as-prepared samples

X-ray diffraction (XRD, Rigaku D/MAX-3B powder diffractometer) with a copper target and K_α_ radiation (λ = 1.54056 Å) was used for the phase identification, where the diffracted X-ray intensities were recorded as a function of 2*θ*. The samples were scanned from 10° to 80° (2*θ*) in steps of 0.02°. Fourier transformed infrared (FTIR) spectra were recorded on AVATAR 360 FT-IR spectrophotometer. The microstructures of the samples were tested on Hitachi S-4800 field emission electron microscopy (FESEM) with the energy dispersive X-ray (EDX) device. The transmission electron micrographs (TEM) were obtained with a Zeiss EM 912 Ω instrument at an acceleration voltage of 120 kV, while high-resolution transmission electron microscopy (HRTEM) characterizations were carried out using a Philips CM200-FEG microscope (200 kV, *C*_s_ = 1.35 mm). The samples used for TEM were prepared by dispersing the products in ethanol with ultrasound treatment, and the dispersion was then dropped on carbon-copper grids. The nitrogen adsorption-desorption technique was measured at 77.3 K with Autosorb iQ Station 1. Prior to the measurement, the sample was degassed at 300 °C for 28.4 h under a vacuum situation. UV-vis measurements were made with a Hitachi U4100 spectrophotometer with a wavelength range between 300 to 700 nm. Photoluminescence (PL) spectra were made with a FLS 980 spectrophotometer of Edinburgh Instruments. X-ray photoelectron spectroscopy (XPS) was carried out at room temperature in ESCALAB 250 system. During XPS analysis, an Al Kα X-ray beam was adopted as the excitation source and the vacuum pressure of the instrument chamber was 1 × 10^−7^ Pa as read on the panel. Measured spectra were decomposed into Gaussian components by a least-square fitting method. Bonding energy was calibrated with reference to C1s peak (285.0 eV).

### Evaluation of the photocatalytic activity of the samples

Since the strong absorption peaks in the visible light, which is favorable the direct evaluation, Rhodamine B (RhB, C_28_H_31_ClN_2_O_3_) is chosen as the model pollutant and indicator of the photocatalytic activity in this work. The photocatalytic properties under visible light of the prepared sample were evaluated with a 24 W visible-light lamp as light source, and the light below 400 nm was removed using a glass filter. In a typical degradation test, 40 mg of the as-synthesized sample was added into a quartz beaker with addition of 50 mL RhB solution (10 mg/L). The distance between the lamp and the solution surface was 6 cm. The dispersion (6 mL) was extracted and centrifuged to separate the catalyst and dye solution at 8500 rev. per min for 10 min at different intervals. The changed RhB concentration of the centrifuged solutions was recorded using a UV-1800 spectrophotometer according to Beer-Lambert’s Law. The UV-vis spectra of the centrifuged solution were also measured using the UV-1800 spectrophotometer from 300 to 600 nm. The photocatalytic performances under sunlight of the g-C_3_N_4_/TiO_2_ were tested through the similar procedures. For the photocatalytic performance of the g-C_3_N_4_/TiO_2_ under sunlight, except that sunlight was directly used as the light source, all other processes are similar with that of visible-light tests. And all the sunlight tests were carried out in sunny days in March 2016 between 11 am and 14 pm in Kunming, China.

### Photoelectrochemical measurements of the samples

The photoelectrochemical measurements were performed on CHI660E (Chenhua Instrument, Shanghai, China) in a neutral aqueous system (0.1 M Na_2_SO_4_) using a three-electrode system. A platinum (Pt) plate and a saturated calomel electrode (SCE, 0.2415 V vs SHE) were used as the counter and the reference electrode, respectively. Light source is provided by 8 W UV lamp (Spectroline EA-180/FE). The working electrode was prepared as follows: 20 mg of the sample was dispersed in 20 mL ethanol with ultrasound for 30 min; 5 μL of the mixture was dropt to an ITO glass (1.25 × 2.5 cm^2^); then the electrode was obtained after drying at 60 °C for 1 h and sintering at 300 °C for 1 h.

## Additional Information

**How to cite this article**: Liu, X. *et al*. A general nonaqueous sol-gel route to g-C_3_N_4_-coupling photocatalysts: the case of Z-scheme g-C_3_N_4_/TiO_2_ with enhanced photodegradation toward RhB under visible-light. *Sci. Rep.* **6**, 39531; doi: 10.1038/srep39531 (2016).

**Publisher's note:** Springer Nature remains neutral with regard to jurisdictional claims in published maps and institutional affiliations.

## Supplementary Material

Supplementary Information

## Figures and Tables

**Table 1:** The *k*, *b* and *R* ^2^ values following the zero-order kinetic process.

Sample	*b*	*k* (h^−1^)	*R*^2^
TiO_2_	0.9484	0.0753	0.9910
2.5% g-C_3_N_4_/TiO_2_	0.8841	0.1021	0.9948
5.0% g-C_3_N_4_/TiO_2_	0.8297	0.1139	0.9942
7.5% g-C_3_N_4_/TiO_2_	0.8924	0.1255	0.9920
10.0% g-C_3_N_4_/TiO_2_	0.8720	0.0656	0.9842
g-C_3_N_4_	0.9820	0.0694	0.9936
P25	0.9308	0.0628	0.9970

## References

[b1] Cao S. W. . g-C_3_N_4_-based photocatalysts for hydrogen generation. J. Phys. Chem. Lett. 5, 2101–2107 (2014). 26270499 10.1021/jz500546b

[b2] Fu J. W. . Dual Z-scheme charge transfer in TiO_2_-Ag-Cu_2_O composite for enhanced photocatalytic hydrogen generation. J. Materiomics 1, 124–133 (2015).

[b3] Akple M. S. . Enhanced visible light photocatalytic H_2_-production of g-C_3_N_4_/WS_2_ composite heterostructures. Appl. Surf. Sci. 358, 196–203 (2015).

[b4] Jin J. . A hierarchical Z-Scheme CdS-WO_3_ photocatalyst with enhanced CO_2_ reduction activity. Small 11, 5262–5271 (2015). 26265014 10.1002/smll.201500926

[b5] Yu W. L. . Enhanced photocatalytic activity of g-C_3_N_4_ for selective CO_2_ reduction to CH_3_OH via facile coupling of ZnO: a direct Z-scheme mechanism. J. Mater. Chem. A 3, 19936–19947 (2015).

[b6] Yu J. G. . Enhanced photocatalytic performance of direct Z-scheme g-C_3_N_4_-TiO_2_ photocatalysts for the decomposition of formaldehyde in air. Phys. Chem. Chem. Phys. 15, 16883–16890 (2013). 23999576 10.1039/c3cp53131g

[b7] Liu Y. N. . Enhanced visible-light photocatalytic activity of Z-scheme graphitic carbon nitride/oxygen vacancy-rich zinc oxide hybrid photocatalysts. Chinese J. Catal. 36, 2135–2144 (2015).

[b8] Zhu C. S. . Fabrication of Z-scheme Ag_3_PO_4_/MoS_2_ composites with enhanced photocatalytic activity and stability for organic pollutant degradation. Appl. Surf. Sci. 377, 99–108 (2016).

[b9] Xiao D. . Hydrothermal synthesis of α-Fe_2_O_3_/g-C_3_N_4_ composite and its efficient photocatalytic reduction of Cr(VI) under visible light. Appl. Surf. Sci. 358, 181–187 (2015).

[b10] Huang S. T. . Z-scheme TiO_2_/g-C_3_N_4_ composites with improved solar-driven photocatalytic performance deriving from remarkably efficient separation of photo-generated charge pairs. Mater. Res. Bull. 84, 65–70 (2016).

[b11] Dong G. H. . Efficient anoxic pollutant removal with oxygen functionalized graphitic carbon nitride under visible light. RSC Adv. 4, 5553–5560 (2014).

[b12] Zhou P. . All-solid-state Z-scheme photocatalytic systems. Adv. Mater. 26, 4920–4935 (2014). 24888530 10.1002/adma.201400288

[b13] Cao S. W. . Polymeric photocatalysts based on graphitic carbon nitride. Adv. Mater. 27, 2150–2176 (2015). 25704586 10.1002/adma.201500033

[b14] Ye S. . A review on g-C_3_N_4_ for photocatalytic water splitting and CO_2_ reduction. Appl. Surf. Sci. 358, 15–27 (2015).

[b15] Wen J. Q. . A review on g-C_3_N_4_-based photocatalysts. Appl. Surf. Sci. 391, 72–123 (2017).

[b16] Hong Y. Z. . *In-situ* synthesis of direct solid-state Z-scheme V_2_O_5_/g-C_3_N_4_ heterojunctions with enhanced visible light efficiency in photocatalytic degradation of pollutants. Appl. Catal. B: Environ. 180, 663–673 (2016).

[b17] Pan C. S. . Dramatic activity of C_3_N_4_/BiPO_4_ photocatalyst with core/shell structure formed by self-assembly. Adv. Funct. Mater. 22, 1518–1524 (2012).

[b18] Yu J. G. . Noble metal-free Ni(OH)_2_-g-C_3_N_4_ composite photocatalyst with enhanced visible-light photocatalytic H-2-production activity. Catal. Sci. Techno. 3, 1782–1789 (2013).

[b19] Pany S. . A facile *in situ* approach to fabricate N, S-TiO_2_/g-C_3_N_4_ nanocomposite with excellent activity for visible light induced water splitting for hydrogen evolution. Phys. Chem. Chem. Phys. 17, 8070–8077 (2015). 25729789 10.1039/c4cp05582a

[b20] Zhou J. W. . Photocatalytic enhancement of hybrid C_3_N_4_/TiO_2_ prepared via ball milling method. Phys. Chem. Chem. Phys. 17, 3647–3652 (2015). 25553728 10.1039/c4cp05173d

[b21] Liao W. J. . Synthesis of Z-scheme g-C_3_N_4_-Ti^3+^/TiO_2_ material: an efficient visible light photoelectrocatalyst for degradation of phenol. Phys. Chem. Chem. Phys. 17, 8877–8884 (2015). 25744448 10.1039/c5cp00639b

[b22] Xu J. . g-C_3_N_4_ modified TiO_2_ nanosheets with enhanced photoelectric conversion efficiency in dye-sensitized solar cells. J. Power Sources 274, 77–84 (2015).

[b23] Li H. . *In situ* growth of TiO_2_ nanocrystals on g-C_3_N_4_ for enhanced photocatalytic performance. Phys. Chem. Chem. Phys. 17, 17406–17412 (2015). 26077198 10.1039/c5cp02554k

[b24] Wang C. . Synthesis of nanocrystalline TiO_2_ in alcohols. Powder Technol. 125, 39–44 (2002).

[b25] Niederberger M. . Benzyl alcohol and transition metal chlorides as a versatile reaction system for the nonaqueous and low-temperature synthesis of crystalline nano-objects with controlled dimensionality. J. Am. Chem. Soc. 124, 13642–13643 (2002). 12431071 10.1021/ja027115i

[b26] Niederberger M. . Organic reaction pathways in the nonaqueous synthesis of metal oxide nanoparticles. Chem. Eur. J. 12, 7282–7302 (2006). 16927442 10.1002/chem.200600313

[b27] Buha J. . Thermal transformation of metal oxide nanoparticles into nanocrystalline metal nitrides using cyanamide and urea as nitrogen source. Chem. Mater. 19, 3499–3505 (2007).

[b28] Deshmukh R. . Ultrasmall Cu_3_N nanoparticles: surfactant-free solution-phase synthesis, nitridation mechanism, and application for lithium storage. Chem. Mater. 27, 8282–8288 (2015).

[b29] Ludi B. . Extension of the benzyl alcohol route to metal sulfides: “nonhydrolytic” thio sol-gel synthesis of ZnS and SnS_2_. Chem. Commun. 47, 5280–5282 (2011). 10.1039/c1cc10856e 21412551

[b30] Pinna N. . Surfactant-free nonaqueous synthesis of metal oxide nanostructures. Angew. Chem. Int. Ed. 47, 5292–5304 (2008). 10.1002/anie.200704541 18561355

[b31] Shi N. . Facile synthesis of monodisperse Co_3_O_4_ quantum dots with efficient oxygen evolution activity. Chem. Commun. 51, 1338–1340 (2015). 10.1039/c4cc08179j 25485907

[b32] Russo P. A. . Room-temperature hydrogen sensing with heteronanostructures based on reduced graphene oxide and tin oxide. Angew. Chem. Int. Ed. 51, 11053–11057 (2012). 10.1002/anie.201204373 23023805

[b33] Baek S. . A one-pot microwave-assisted non-aqueous sol-gel approach to metal oxide/graphene nanocomposites for Li-ion batteries. RSC Adv. 1, 1687–1690 (2011).

[b34] Russo P. A. . Microwave-assisted coating of carbon nanostructures with titanium dioxide for the catalytic dehydration of D-xylose into furfural. RSC Adv. 3, 2595–2603 (2013).

[b35] Jensen G. V. . Anisotropic crystal growth kinetics of anatase TiO_2_ nanoparticles synthesized in a nonaqueous medium. Chem. Mater. 22, 6044–6055 (2010).

[b36] Niederberger M. . Benzyl alcohol and titanium tetrachlorideA versatile reaction system for the nonaqueous and low-temperature preparation of crystalline and luminescent titania nanoparticles. Chem. Mater. 14, 4364–4370 (2002).

[b37] Niederberger M. . Tailoring the surface and solubility properties of nanocrystalline titania by a nonaqueous *in situ* functionalization process. Chem. Mater. 16, 1202–1208 (2004).

[b38] Zhu J. . Nanocrystalline anatase TiO_2_ photocatalysts prepared via a facile low temperature nonhydrolytic sol-gel reaction of TiC1(4) and benzyl alcohol. Appl. Catal. B: Environ. 76, 82–91 (2007).

[b39] Dong G. H. . Carbon self-doping induced high electronic conductivity and photoreactivity of g-C_3_N_4_. Chem. Comm. 48, 6178–6180 (2012). 22588283 10.1039/c2cc32181e

[b40] Dong G. H. . Porous structure dependent photoreactivity of graphitic carbon nitride under visible light. J. Mater. Chem. 22, 1160–1166 (2012).

[b41] Zhu B. C. . Fabrication and photocatalytic activity enhanced mechanism of direct Z-scheme g-C_3_N_4_/Ag_2_WO_4_ photocatalyst. Appl. Surf. Sci. http://dx.doi.org/10.1016/j.apsusc.2016.07.104 (2016).

[b42] Zhu B. C. . Isoelectric point and adsorption activity of porous g-C_3_N_4_. Appl. Surf. Sci. 344 188–195 (2015).

[b43] Xu L. L. . Nano-MnO_x_ on activated carbon prepared by hydrothermal process for fast and highly efficient degradation of azo dyes. Appl. Catal. A: Gen. 485, 91–98 (2014).

[b44] Chen W. . *In situ* fabrication of novel Z-scheme Bi_2_WO_6_ quantum dots/g-C_3_N_4_ ultrathin nanosheets heterostructures with improved photocatalytic activity. Appl. Surf. Sci. 355, 379–387 (2015).

[b45] Zhou D. T. . *In-situ* construction of all-solid-state Z-scheme g-C_3_N_4_/TiO_2_ nanotube arrays photocatalyst with enhanced visible-light-induced properties. Sol. Energ. Mat. Sol. C. 157, 399–405 (2016).

[b46] Wen J. Q. . Photocatalysis fundamentals and surface modification of TiO_2_ nanomaterials. Chinese J. Catal. 36, 2049–2070 (2015).

[b47] Liu H. R. . Worm-like Ag/ZnO core-shell heterostructural composites: fabrication, characterization, and photocatalysis. J. Phys. Chem. C 116, 16182–16190 (2012).

[b48] Ma J. Z. . Enhanced photocatalytic oxidation of NO over g-C_3_N_4_-TiO_2_ under UV and visible light. Appl. Catal. B: Environ. 184, 28–34 (2016).

[b49] Li Y. L. . Seed-induced growing various TiO_2_ nanostructures on g-C_3_N_4_ nanosheets with much enhanced photocatalytic activity under visible light. J. Hazard. Mater. 292, 79–89 (2015). 25797926 10.1016/j.jhazmat.2015.03.006

[b50] Lv J. L. . Facile synthesis of Z-scheme graphitic-C_3_N_4_/Bi_2_MoO_6_ nanocomposite for enhanced visible photocatalytic properties. Appl. Surf. Sci. 358, 377–384 (2015).

[b51] Li J. Q. . Improved photoelectrochemical performance of Z-scheme g-C_3_N_4_/Bi_2_O_3_/BiPO_4_ heterostructure and degradation property. Appl. Surf. Sci. 385, 34–41 (2016).

[b52] Luo J. . Rational construction of Z-scheme Ag_2_CrO_4_/g-C_3_N_4_ composites with enhanced visible-light photocatalytic activity. Appl. Surf. Sci. 390, 357–367 (2016).

[b53] Han J. H. . AgSbS_2_ modified ZnO nanotube arrays for photoelectrochemical water splitting, Appl. Catal. B: Environ 179, 61–68 (2015).

[b54] Wu W. Q. . Multistack intergration of three-dimensional hyperbranched anatase titania architectures for high-efficiency dye-sensitized solar cells. J. Am. Chem. Soc. 136, 6437–6445 (2014). 24725076 10.1021/ja5015635

[b55] Hsu S. C. . Modulation of photocarrier dynamics in indoline dye-modified TiO_2_ nanorod array/P3HT hybrid solar cell with 4-tert-butylpridine. J. Phys. Chem. C 116, 25721–25726 (2012).

[b56] Wang Y. J. . Enhancement of photocurrent and photocatalytic activity of ZnO hybridized with graphite-like C_3_N_4_. Energy Environ. Sci. 4, 2922–2929 (2011).

[b57] Xu D. F. . Enhanced photocatalytic activity and stability of Z-scheme Ag_2_CrO_4_-GO composite photocatalysts for organic pollutant degradation. Appl. Catal. B: Environ. 164, 380–388 (2015).

[b58] Rehman S. . Strategies of making TiO_2_ and ZnO visible light active. J. Hazard. Mater. 170, 560–569 (2009). 19540666 10.1016/j.jhazmat.2009.05.064

[b59] Isao N. . Role of oxygen vacancy in the plasma-treated TiO_2_ photocatalyst with visible light activity for NO removal. J. Mol. Catal. A: Chem. 161, 205–212 (2000).

[b60] Ihara T. . Visible-light-active titanium oxide photocatalyst realized by an oxygen-deficient structure and by nitrogen doping. Appl. Catal. B: Environ. 42, 403–409 (2003).

[b61] Chen N. . Xylene sensing performance of WO_3_ decorated anatase TiO_2_ nanoparticles as a sensing material for gas sensor at low operating temperature. RSC Adv. 6, 49692–49701 (2016).

[b62] Kumar S. . Cost-effective and eco-friendly synthesis of novel and stable N-doped ZnO/g-C_3_N_4_ core-shell nanoplates with excellent visible-light responsive photocatalysis. Nanoscale 6, 4830–4842 (2014). 24664127 10.1039/c3nr05271k

[b63] Jo W. K. . Influence of TiO_2_ morphology on the photocatalytic efficiency of direct Z-scheme g-C_3_N_4_/TiO_2_ photocatalysts for isoniazid degradation. Chem. Eng. J. 281, 549–565 (2015).

[b64] Moon J. . Photocatalytic activation of TiO_2_ under visible light using Acid Red 44. Catal. Today 87, 77–86 (2003).

[b65] Zhao J. C. . Photocatalytic degradation of organic pollutants under visible light irradiation. Top. Catal. 35, 269–278 (2005).

[b66] Liu X. . One-step nonaqueous sol-gel route to mixed-phase TiO_2_ with enhanced photocatalytic degradation of Rhodamine B under visible light. CrystEngComm 18, 1964–1975 (2016).

[b67] Shalom M. . Improving carbon nitride photocatalysis by supramolecular preorganization of monomers. J. Am. Chem. Soc. 135, 7118–7121 (2013). 23647353 10.1021/ja402521s

