# Supplementary material for: A general nonaqueous sol-gel route to g-C3N4-coupling photocatalysts: the case of Z-scheme g-C3N4/TiO2 with enhanced photodegradation toward RhB under visible-light
Source: Sci Rep. 2016 Dec 22;6:39531. doi: 10.1038/srep39531 (PMC5177904; doi:10.1038/srep39531)
Supplement: Supplementary Information [file srep39531-s1.doc]

**Supplementary Information**

**A general nonaqueous sol-gel route to** **g-C_3_N_4_-coupling photocatalysts: The case of Z-scheme g-C_3_N_4_/TiO_2_ with enhanced photodegradation toward RhB under visible-light**

Xu Liu ^a^, Nan Chen ^b^, Yuxiu Li ^a^, Dongyang Deng ^a^, Xinxin Xing ^a^, Yude Wang^*b,c^

a School of Materials Science and Engineering, Yunnan University, 650091 Kunming, People’s Republic of China

b Department of Physics, Yunnan University, 650091 Kunming, People’s Republic of China

c Yunnan Province Key Lab of Micro-Nano Materials and Technology, Yunnan University, 650091, Kunming, People’s Republic of China

**Table S1.** Structural data and refinement parameters of TiO_2_ nanoparticles calculated by Rietveld refinement of the experimental XRD powder pattern.

| Space group | *I*4_1_/*amd* (141) |
| --- | --- |
| Lattice parameters |  |
| *a* (Å) | 3.7926 |
| *b* (Å) | 3.7926 |
| *c* (Å) | 9.5111 |
| Ti |  |
| *x* | 0 |
| *y* | 0 |
| *z* | 0 |
| O |  |
| *x* | 0 |
| *y* | 0 |
| *z* | 0.2126 |
| Average crystallite size (nm) | 7.65 |
| Average maximum strain (10^-3^*)* | 3.18 |
| *R*_WP_ (%) | 28.52 |
| *R*_P_ (%) | 21.11 |


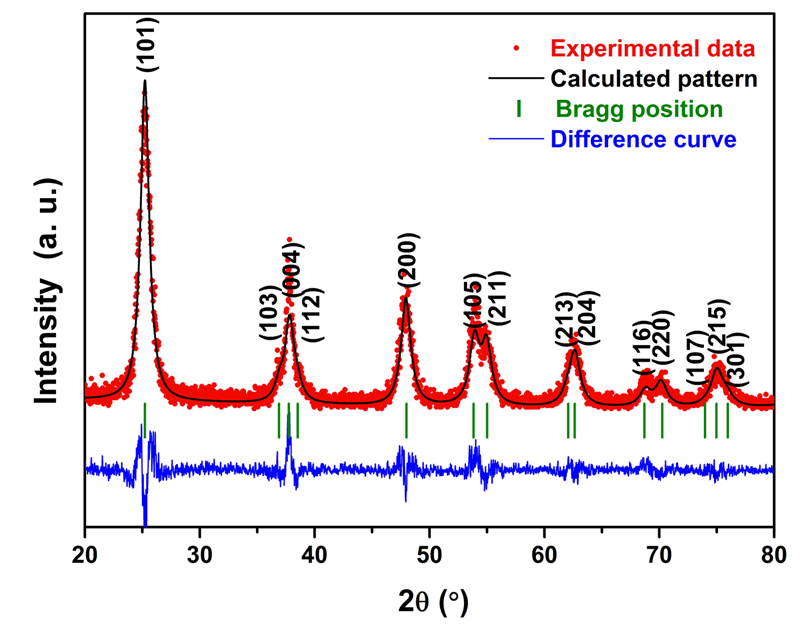


**Figure S1** Typical Rietveld output plot of as-prepared TiO_2_ nanoparticles. The experimental data, calculated pattern and the difference curve are shown in red, black and blue, respectively. The short green bars in green represent the positions of Bragg reflections.

**Figure S2** FESEM images of as-prepared (a) 2.5%, (b) 7.5% and (c) 10% g-C_3_N_4_/TiO_2_.


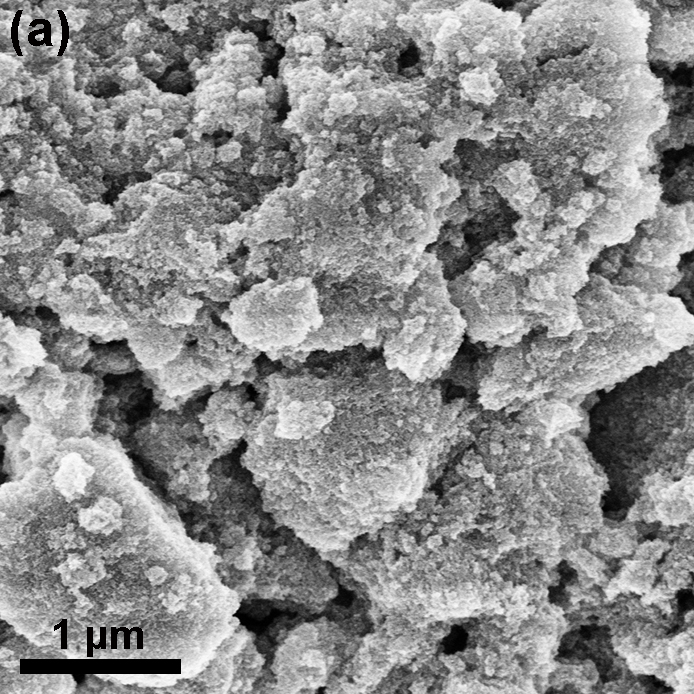

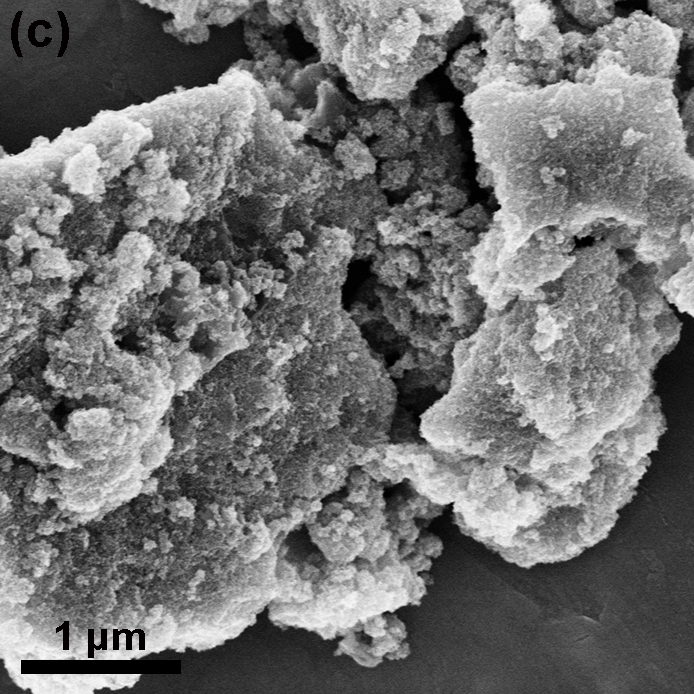

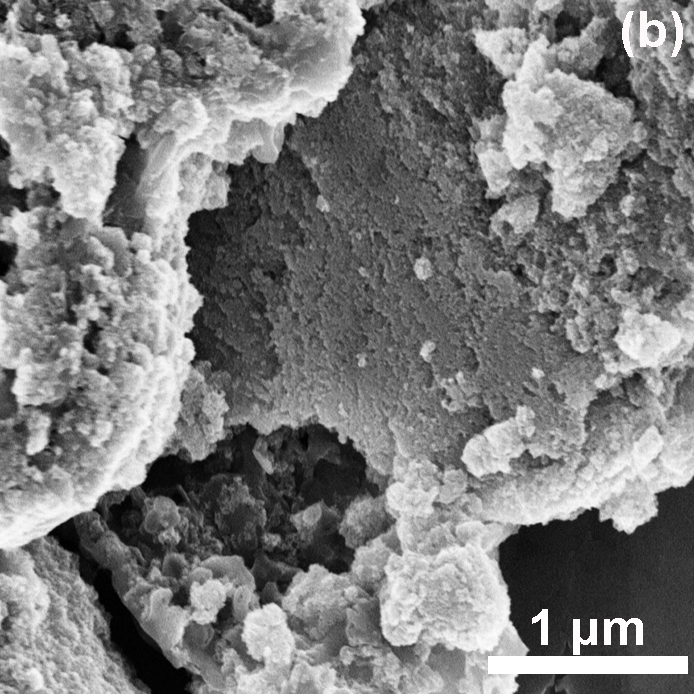


**
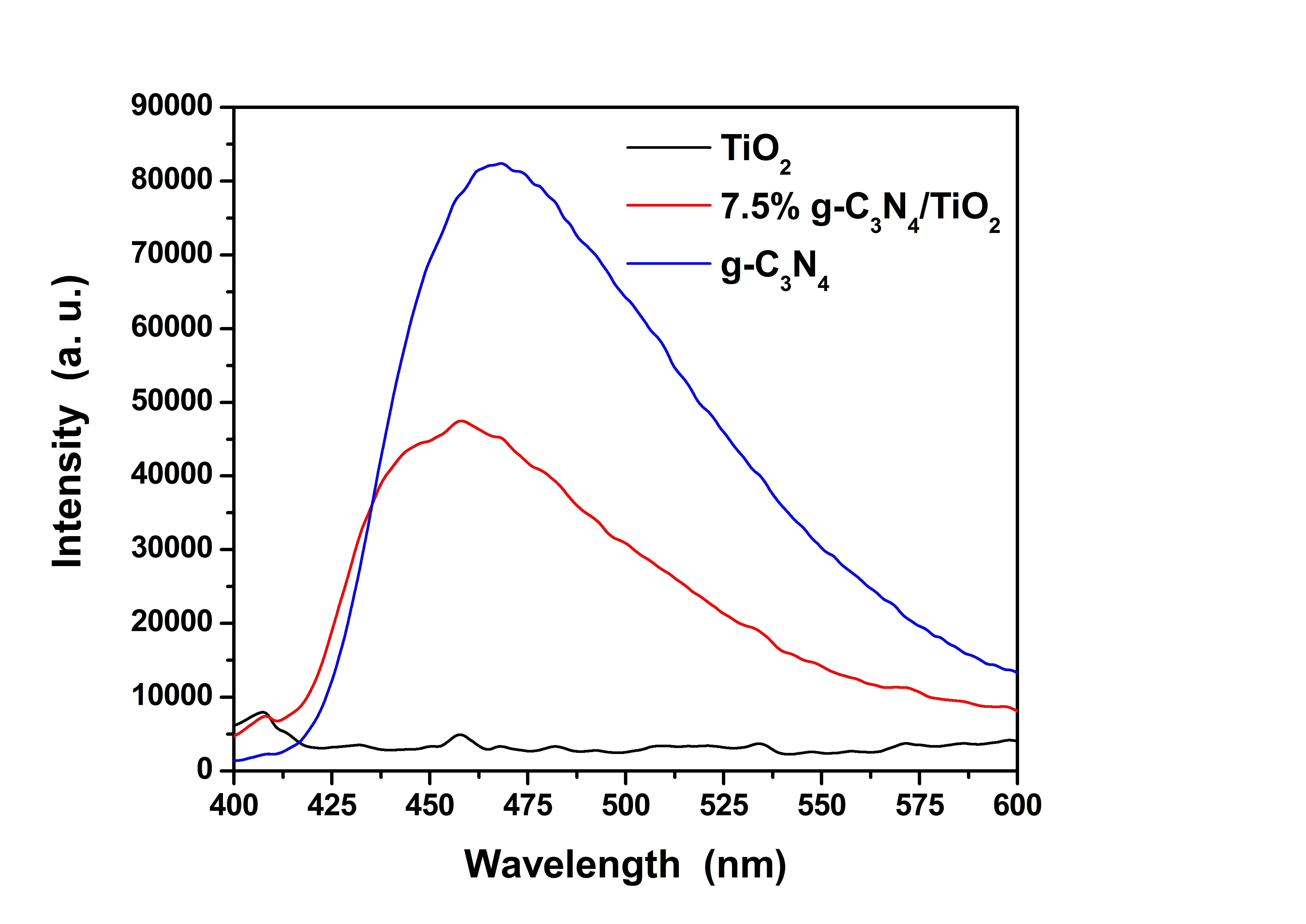
**

**Figure S3** Photoluminescence spectra of g-C_3_N_4_, TiO_2_ and 7.5% g-C_3_N_4_/TiO_2_ with an excited wavelength of 325 nm.

**
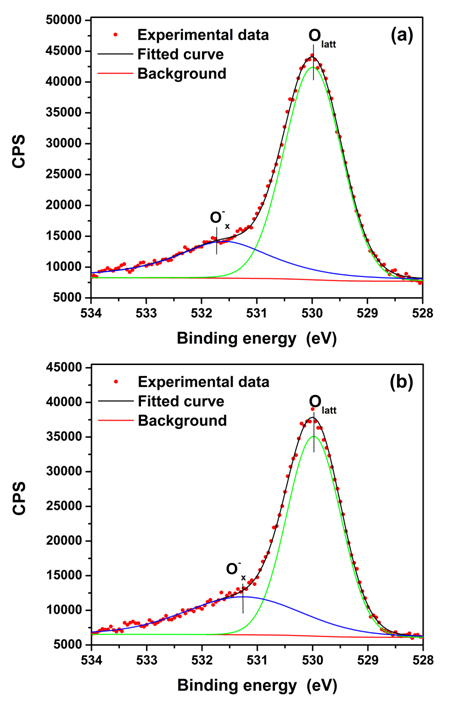
**

**Figure S4** O1s XPS spectra of as-prepared TiO_2_ and 7.5% g-C_3_N_4_/TiO_2_.

**
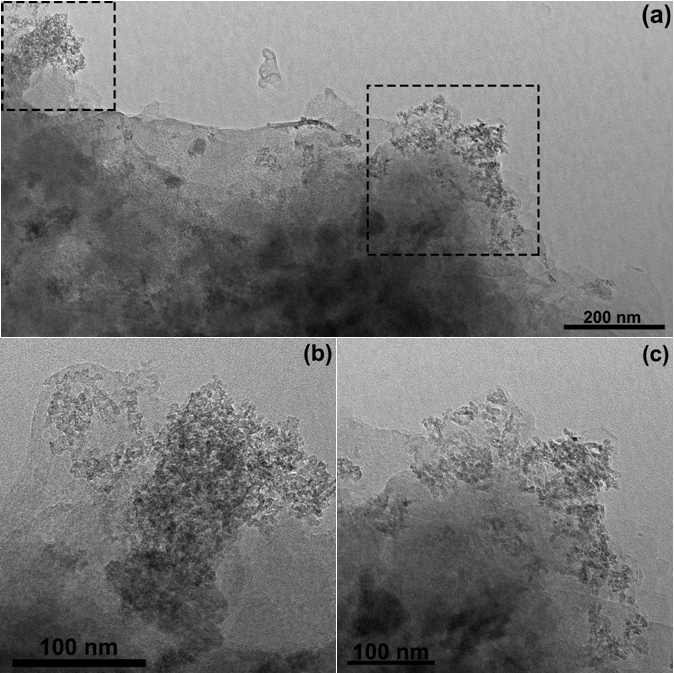
**

**Figure S5** TEM imagines of 7.5% g-C_3_N_4_/TiO_2_ after five repeated photocatalytic experiments under irradiation of sunlight.
